# Endothelial cell dysfunction in viral hemorrhage and edema

**DOI:** 10.3389/fmicb.2014.00733

**Published:** 2015-01-05

**Authors:** Erich R. Mackow, Elena E. Gorbunova, Irina N. Gavrilovskaya

**Affiliations:** Department of Molecular Genetics and Microbiology, Stony Brook University, Stony Brook, NY, USA

**Keywords:** endothelial, hantavirus, dengue, arenavirus, PD-L1, platelet, permeability barrier, tolerance induction

## Abstract

The endothelium maintains a vascular barrier by controlling platelet and immune cell interactions, capillary tone and interendothelial cell (EC) adherence. Here we suggest common elements in play during viral infection of the endothelium that alter normal EC functions and contribute to lethal hemorrhagic or edematous diseases. In viral reservoir hosts, infection of capillaries and lymphatic vessels may direct immunotolerance without disease, but in the absence of these cognate interactions they direct the delayed onset of human disease characterized by thrombocytopenia and vascular leakage in a severe endothelial dysfunction syndrome. Here we present insight into EC controls of hemostasis, immune response and capillary permeability that are altered by viral infection of the endothelium.

The role of the endothelium in maintaining fluid barrier functions of capillaries is clear, obvious and fundamental to virally induced hemorrhagic and edematous disease. Endothelial cells (ECs) control vascular permeability through a dynamic set of “fail-safe” systems. ECs permit fluid and cellular efflux without leakage, yet respond to diverse signals from tissues and the circulation, and elicit responses that regulate platelets, complement and immune cell activation as well as capillary dilation and permeability (Luscher and Barton, 1997; Willoughby and Loscalzo, 2002; Orfanos et al., 2004; Aird, 2008; Dvorak, 2010). However, it is often overlooked that EC receptors, signaling pathways and activation responses are as distinct and varied as those of T and B cells. In fact, EC signaling pathways are uniquely wired to avoid apoptosis, T cell targeting and disruption of the endothelium. The checks and balances built into averting lethal capillary leakage suggest that several EC controls are bypassed by viruses that cause hemorrhagic and edematous disease.

Except for the outcome, the mechanisms of vascular dysfunction in virally induced capillary leakage are poorly understood. Viral infection of the endothelium is difficult to study and by default roles for capillary leakage are consigned to more easily assessed blood constituents. In viral infections the vasculature is largely considered a conduit and secondary to the circulating cytokines, suggested to form a “cytokine storm” that acts on the endothelium. Yet, if cytokine responses were the cause of vascular leakage, a wide variety of viruses, like influenza virus, should be hemorrhagic (Teijaro et al., 2011). Similar patient cytokine responses are observed during dengue fever (DF), that occurs in the absence of hemorrhage or edema, and in patients that develop dengue hemorrhagic fever (DHF) and dengue shock syndrome (DSS; Halstead, 2002, 2008). In fact, while many viruses cause a systemic cytokine storm there are only a few viruses that cause hemorrhagic disease. Opinions are changing on the role of the cytokine storm in virology, sepsis and autoimmune diseases since the same cytokines are recorded without vascular collapse or shock. An expanded, paradigm referred to as “severe endothelial dysfunction syndrome” has been forwarded by focus groups at the National Heart Lung and Blood Institute to more accurately reflect viral and bacterial vascular leak syndromes (http://www.nhlbi.nih.gov/research/reports/2010-bsrts.htm).

What differentiates viruses that cause hemorrhage, edema and shock from others? Infection of the endothelium combined with thrombocytopenia, capillary permeability and the control of immune cells, directly or by tolerance, are high on a list of EC regulated responses associated with virally induced vascular leakage.

## ENDOTHELIAL CELL REGULATED TOLERANCE AND VIRAL HEMORRHAGE DISEASE

Viruses that rapidly infect and spread cause acute self-limited infections that are in general cleared by immune responses without causing thrombocytopenia or vascular leakage. Herpes viruses and non-hemorrhagic arenaviruses (AVs) infect immune and ECs without thrombocytopenia or capillary leakage and establish persistent viral infections, in humans or hosts, by directing immunotolerance (Adler and Sinzger, 2009; Kunz, 2009). In contrast, AVs that cause hemorrhagic or edematous disease (Sabia, Lassa, Mopeia, Junin, etc.) induce thrombocytopenia 1–2 weeks post-infection and fail to tolerize humans, but persistently and asymptomatically infect their small mammal hosts (Kunz, 2009; Charrel and de Lamballerie, 2010; Moraz and Kunz, 2011). Ebola virus (EBOV) also infects human immune, dendritic and ECs causing thrombocytopenia, lymphopenia and immune and EC dysfunction 1–3 weeks after inoculation (Chen and Cosgriff, 2000; Schnittler and Feldmann, 2003; Aleksandrowicz et al., 2008). Similar to other viral-host infections, EBOV presumably establishes persistence in its infected reservoir hosts (possibly fruit bats), without known disease, and it can be reasoned that EBOV attempts tolerizing humans and subsequently causes immune, platelet and EC dysfunction (Schnittler and Feldmann, 2003; Mohamadzadeh et al., 2007). EBOV and AVs have in common their ability to asymptomatically establish persistent infections of hosts despite infecting immune and ECs. In humans both viruses cause vascular leakage characterized by a long onset period prior to hemorrhagic or edematous disease and are resistant to interferon and replication inhibitors when patients are symptomatic. These findings are consistent with failed attempts to establish persistence in humans. Inactivation of the immune system may also contribute to an extended period of EC infection that alters the delicate balance of EC regulated hemostasis.

## ROLE FOR PD-L1/PD-1 RESPONSES IN CAPILLARY PERMEABILITY

Tolerance can be mediated by immune or EC responses and many excellent reviews address T cell exhaustion and regulation (Francisco et al., 2010; Jin et al., 2011; Kamphorst and Ahmed, 2013; Penaloza-MacMaster et al., 2014). In persistent AV [lymphocytic choriomeningitis virus (LCMV)] infections tolerance is the result of suppressive PD-L1 (CD274) signals that engage T cell expressed PD-1 (CD279) receptors, limiting effector T cell clearance and causing T cell anergy (Mueller et al., 2010; Wei et al., 2013; Penaloza-MacMaster et al., 2014). Although the role of EC-derived PD-L1 has not been studied during AV infections, PD-L1 inhibits T cell targeting, lysis and immunopathology but may also play a unique role in tolerance that is linked to EC permeability (Rodig et al., 2003; Brooks et al., 2008; Tewalt et al., 2012b). A pointed demonstration of PD-L1/PD-1 responses in hemorrhagic and edematous diseases is provided by findings that LCMV infection of PD-1 knockout mice resulted in a dramatic increase in pulmonary edema as well as increased edema in brain, liver and kidney (Frebel et al., 2012). Consistent with this, pulmonary edema was also observed in wt LCMV infected mice treated with a blocking antibody to PD-L1 (Frebel et al., 2012). PD-1 KOs had increased plasma thrombin-antithrombin III (ATIII) levels and a 90% reduction in platelet levels, further suggesting a role for dysregulated PD-L1/PD-1 responses in thrombocytopenia during viral infections.

Less is known about EC mediated tolerance and its potential role in viral hemorrhagic and edematous disease (Rodig et al., 2003; Tewalt et al., 2012a,b). Microvascular and lymphatic ECs (MECs and LECs) regulate vessel integrity and fluid clearance functions of lymphatics (Aird, 2008; Schraufnagel, 2010). However, they also serve as non-professional antigen presenting cells that line lymphoid tissues and capillaries and are reportedly essential for establishing tolerance to antigens present within the endothelium (Aird, 2008; Schraufnagel, 2010; Tewalt et al., 2012a,b). EC induced tolerance restricts T cell targeting of antigens within the endothelium and at one level controls immune mediated vascular leakage (Tewalt et al., 2012a,b). This ploy appears to be engaged by several viruses that induce persistence, but with the wrong viral protein repertoire, attempted tolerance may instead increase the duration of EC infection and uncouple normally balanced hemostatic functions.

The role of the endothelium in tolerance and vascular permeability is simplified in hantavirus (HV) infections where HVs nearly exclusively replicate in ECs of humans and hosts (Zaki et al., 1995; Mackow et al., 2013; Vaheri et al., 2013). HVs persistently infect their small mammal hosts without causing disease, but like AV and EBOV cause thrombocytopenia and vascular leakage 1–3 weeks after infection of humans (Gavrilovskaya et al., 1990; Zaki et al., 1995). Although HVs infect ECs throughout the body they cause overt disease in expansive EC capillary beds of the lungs and kidneys resulting in hantavirus pulmonary syndrome (HPS; Duchin et al., 1994; Zaki et al., 1995; Bustamante et al., 1997; Koster and Mackow, 2012) and hemorrhagic fever with renal syndrome (HFRS; Cosgriff and Lewis, 1991; Lahdevirta, 1982).

A compelling case is emerging for HVs to induce EC directed T cell regulation and tolerizing responses that contribute to disease. MECs and LECs express inhibitory PD-L1 receptors on their surfaces that suppress CD4^+^ and CD8^+^ T-cell responses and induce tolerance (Rodig et al., 2003; Tewalt et al., 2012a,b). PD-L1/PD-1 responses form a fundamental immune regulatory paradigm that controls CD4^+^ T cell exhaustion and is resolved by a PD-L1 blockade (Francisco et al., 2009, 2010; Penaloza-MacMaster et al., 2014). During acute human HV infections that cause HPS, ECs in the lung are uniformly and singularly infected resulting in acute pulmonary edema with exudates accumulating at up to 1 l per hour (Zaki et al., 1995; Bustamante et al., 1997). In HPS, immunoblasts (>10%) are a feature of infection and present at high levels in lymphoid organs and concentrated in the lung at autopsy (Zaki et al., 1995). Its unclear whether HV infection of pulmonary ECs tolerizes or arrests immunoblasts (Zaki et al., 1995) or observed T cell responses (Ennis et al., 1997; Kilpatrick et al., 2004), but even at autopsy there is little evidence of endothelial cytopathic effect during HPS and no hemorrhagic disease indicative of a capillary breech (Zaki et al., 1995). While regulatory CD4 or CD8 T cell responses were not observed during HFRS infections, activated CD4 and CD8 T cells were observed during human Puumala virus (HV) infections (Lindgren et al., 2011). However, Puumala virus causes more of an early acute infection and there is no indication of the T cell responses relative to disease (Terajima et al., 2007; Lindgren et al., 2011). Even in this setting CD4 and CD8 T cells were found to express inhibitory receptors (PD-1, CTLA-4, or both) that may balance effector functions with tolerance (Lindgren et al., 2011). Consistent with HPS responses in patients, both hypoxia and interferons induce the expression of PD-L1 (Schreiner et al., 2004; Muhlbauer et al., 2006; Noman et al., 2014). The Seoul HV reportedly increases PD-L1 expression levels during in vitro infection of ECs (Li and Klein, 2012) and our recent findings indicate that pathogenic HV infection (ANDV, HPS), but not infection by non-pathogenic TULV, upregulates PD-L1 levels on the surface of ECs. These findings may link EC expressed PD-L1 to the increased duration of viral infections of the endothelium that may contribute to EC dysfunction (Mueller et al., 2010). These findings also suggest a mechanism for HV infected ECs to inhibit T cell responses via PD-L1/PD-1 regulation.

Hantavirus induced T cell exhaustion could also explain why T cell depletion therapy was found to have no effect on the timing, onset or severity of lethal HPS disease in ANDV infected Syrian hamsters, and why steroid immunosuppression actually permits SNV directed HPS in this model (Hammerbeck and Hooper, 2011; Brocato et al., 2014). These finding suggest that T cell responses may already be suppressed by ANDV infection and that immune mediated disease isn’t a likely cause of vascular permeability in HPS. In a lethal non-human primate HPS model, T cell responses and inflammatory cytokines were absent 12 dpi even though radiographic pulmonary edema and cardiac enlargement were observed 6–9 and 12 dpi, respectively, and prior to T cell and cytokine responses (first noted 18–21 dpi; Safronetz et al., 2014). Although it was not determined whether T cells were PD-1 or CTLA-4 positive, HVs fail to establish persistence in humans and tolerizing responses may alter the timing, vigor and targeting of T cell responses that normally clear infection (Terajima et al., 2007; Mueller et al., 2010). The extended HV infection of MECs and LECs may also dysregulate functions of the endothelium that control: platelet activation and recruitment (Raymond et al., 2005; Sohn et al., 2005; Gavrilovskaya et al., 2010), EC responses to a plethora of potential permeabilizing factors (Nagy et al., 2008; Dvorak, 2010; Gorbunova et al., 2010; Taylor et al., 2013), fluid clearance from tissues by lymphatic vessels and capillary tone (Luscher and Barton, 1997; Yang et al., 2004; Aird, 2008; Gavrilovskaya et al., 2012; Lin et al., 2013). Given the delicate balance of hemostatic factors regulated by ECs, it is unclear whether targeting platelets, ECs or *β*3 integrin receptors, or effecting a PD-L1 blockade/Il-2 stimulation to restore viral clearance, would be therapeutic or deleterious to the endothelium and lethal capillary leakage (Kamphorst and Ahmed, 2013; West et al., 2013).

## ENDOTHELIAL CELL FUNCTIONS

**FIGURE 1:**
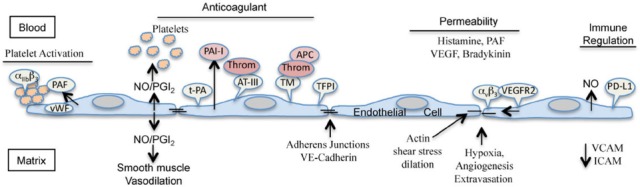
**Endothelial cell functions that maintain hemostasis.** Endothelial cells are primary anticoagulants that prevent platelet and immune cell adherence and restrict the activation of clotting cascades (Hoffman and Monroe, 2001; Feletou, 2011). ECs display tissue plasminogen activator (t-PA) but constitutively secrete plasminogen activator inhibitor type I (PAI-I). ECs constitutively express tissue factor pathway inhibitor (TFPI) on their surfaces that counteracts tissue factor activated coagulation. ECs regulate thrombin (Throm) activation by presenting anti-thrombin-III (AT-III) and thrombomodulin (TM) on their surfaces which respectively bind and inactivate thrombin or direct thrombin activation of protein C (APC). ECs express eNOS which directs the production of nitric oxide and secrete prostaglandin I_2_ (PGI_2_, prostacyclin) that both inhibit platelet activation and aggregation and are potent vasodilators that increase blood flow (Pober and Sessa, 2007). However, ECs also express von Willebrand factor within storage granules and secrete the platelet activating factor that activate platelets. ECs restrict responses of immune cells by expressing the T-cell activation inhibitor PD-LI on their surfaces and secreting NO and by preventing the expression of immune recruiting receptors like intercellular cell adhesion molecule-1 (ICAM) or vascular cell adhesion molecule (VCAM). ECs constitutively express α_v_β_3_ integrins on their surface that are required for EC migration and regulate vascular endothelial growth factor receptor 2 (VEGFR2) responses to the VEGF, a potent edemagenic factor, first discovered as a permeability factor. Hypoxia, angiogenesis and extravasation of immune cells requires dissociation of adherens junctions in order to restore tissue oxygenation and effect EC migration and vascular repair. These processes and responses to histamine, platelet activating factor VEGF, bradykinin along with shear stress and dilatory stretching of the endothelium can increase vascular permeability and are regulated by circulating soluble receptors or degradative systems which normally limit VEGF and bradykinin effects in order to maintain hemostasis. Viral infection of the endothelium has the potential to alter the delicate balance of these interrelated systems and signals to direct fibrinoloysis and disseminated intravascular coagulation, activate permeability pathways, alter platelet responses, and control immune cell recognition of the endothelium. Each virus mentioned appears to perturb these functions in unique ways that are enhanced by prolonged infection of ECs, platelet dysfunction and failed immune clearance. A more complete analysis of hemostatic control mediated by ECs and extensive figures on the subject are present in several excellent reviews (Luscher and Barton, 1997; Hoffman and Monroe, 2001; Aird, 2004, 2008; Pober and Sessa, 2007; Martina et al., 2009; Feletou, 2011).

Several excellent reviews detail the wealth of systems at play in the endothelium and their dynamic management of capillary functions (Luscher and Barton, 1997; Willoughby and Loscalzo, 2002; Orfanos et al., 2004; Nagy et al., 2008; Pober et al., 2009). Under normal conditions, the endothelium constitutively directs the inactivation of platelets, clotting and complement cascades and prevents the adherence of immune cells and platelets to ECs (Figure 1). However, ECs also dynamically respond to conditions in order to activate platelets, secrete chemokines, recruit and extravasate immune cells and control vessel dilation and repair (Aird, 2008). This is accomplished through a combination of EC specific proliferative responses, receptors and permeability factors that both act on the endothelium or are secreted or expressed by ECs.

## EC DIRECTED HEMOSTASIS: PLATELETS, COAGULATION, VESSEL TONE, AND PERMEABILITY

A detailed understanding of hemostasis is beyond the scope and focus of this paper but several excellent reviews address these interrelated responses that are controlled by ECs (Hoffman and Monroe, 2001; Aird, 2008; Pober et al., 2009; Dvorak, 2010; Feletou, 2011). Hemostasis is maintained by EC interactions with platelets and regulation of fibrin formation. The endothelium is normally quiescent and does not recruit platelets or immune cells or direct fibrinolysis or coagulation. ECs are dedicated to remaining inert and preserving this balance, however disrupting the endothelium exposes subendothelial matrix that directs platelet activation and adherence through integrin receptors. ECs store and release von Willebrand factor (vWF) which stabilizes Factor VIII and furthers platelet receptor interactions (Hoffman and Monroe, 2001). ECs also synthesize and release platelet activating factor (PAF) that further promotes platelet adherence to ECs. Tissue factor (TF) is a cell associated coagulation activator that is present in the extracellular matrix and initiates coagulation cascades following exposure of blood to the tissue. TF initiates a cascade that results in the generation of thrombin and the cleavage of fibrinogen to fibrin which aggregates platelets.

Several EC directed mechanisms inhibit coagulation in order to prevent platelet adhesion and aggregation (Hoffman and Monroe, 2001; Pober and Sessa, 2007; Pober et al., 2009; Feletou, 2011). ECs express TF pathway inhibitor (TFPI) on their surfaces which prevents TF directed coagulation and present ATIII on their surfaces which inactivates thrombin. ECs highly express thrombomodulin (TM) on their surfaces which binds thrombin and converts it to an anticoagulant by binding and activating protein C (Figure 1). Activated protein C binds protein S and inactivates factor Va and VIIIa. ECs normally mask signals that recruit platelets and express prostaglandin I_2_ (prostacyclin) and nitric oxide (NO) which both inhibit platelet adhesion and direct vasodilation that increases blood flow (Hoffman and Monroe, 2001; Pober and Sessa, 2007; Pober et al., 2009; Feletou, 2011).

Fibrinolysis is also EC regulated since ECs constitutively synthesize tissue-type plasminogen activator (t-PA) and induce t-PA in response to shear stress, thrombin and bradykinin (Feletou, 2011). However, this activity is controlled by plasminogen activator inhibitor type I (PAI-I) that is also constitutively secreted by ECs. Collectively these EC regulated responses control coagulation, dilation, fibrinolysis and thrombosis that may be altered during viral infection of the endothelium resulting in aberrant coagulation and hemostatic responses that contribute to disseminated intravascular coagulation (DIC), hemorrhage and edema (Figure 1).

Endothelial barrier functions can be altered by many of the components above that control hemostasis by directing changes in inter-EC junctions and result in edema or hemorrhage. Mediators of EC permeability include histamine, serotonin thrombin, bradykinin, PAF, cytokines and several growth factors including EC specific vascular endothelial growth factor (VEGF) that direct angiogenesis and repair (Dvorak, 2006, 2010; Feletou, 2011). Hypoxic conditions within tissues direct vascular permeability and vessel repair which by necessity loosens EC connections to permit EC migration. Edema results from an excess of fluid in the tissues relative to the ability of lymphatic vessels to remove interstitial fluid (Figure 1). Not surprisingly, these overlapping functions and feedback mechanisms, which are in place to prevent lethal vascular leakage, are altered by viral infection of ECs and fundamental to severe endothelial dysfunction syndromes directed by viruses.

## VIRAL REGULATION OF ENDOTHELIAL SIGNALING PATHWAYS

Successful viral infections are accompanied by the regulation of Type I interferon (in ECs IFN*β*) responses that are present to innately control their replication. AVs, EBOVs, HVs, and dengue virus (DV) all regulate signaling pathways that induce IFN induction or cellular responses to IFN receptor activation (Alff et al., 2006; Rodriguez-Madoz et al., 2010; Luthra et al., 2013; Cimica et al., 2014). But late in infection IFN responses are induced by all these viruses. However, both the induction of IFN and cellular responses to exogenous IFN are unique in ECs. In contrast to immune cells which apoptotically respond to IFN, ECs treated with type I IFN proliferate (Gomez and Reich, 2003), protecting and enhancing barrier functions of the endothelium following viral infections. These findings suggest that early viral inhibition of IFN secretion and later IFN induction may positively or negatively impact vascular permeability of the virally infected endothelium (Schreiner et al., 2004). The protective role of IFN on the endothelium questions the use of IFN receptor knockouts to study the pathogenesis of virally induced vascular hemorrhagic and edematous disease (Williams et al., 2009; Zust et al., 2014).

HV proteins inhibit IFN induction by binding TRAF3, and blocking TBK1 directed responses (Alff et al., 2008; Cimica et al., 2014; Matthys et al., 2014). Similar pathway targeting occurs in AV, EBOV, and DV infections (Rodriguez-Madoz et al., 2010; Pythoud et al., 2012; Rodrigo et al., 2012; Luthra et al., 2013). TBK1 and TRAF3 are tied to NF-*κ*B and VEGF/hypoxia to mTOR signaling pathways that control EC survival, growth factor, cytokine, hypoxia, and hemostatic responses (Venkatesha et al., 2004; Sohn et al., 2005; Madge and May, 2010; Hacker et al., 2011; Laplante and Sabatini, 2012). Curiously, TBK1, which is normally expressed at catalytic levels in most cells (Hacker et al., 2011; Tu et al., 2013), is expressed at high levels in primary human ECs. Although the significance of this remains to be determined, these findings point to the novel wiring and regulation of signaling pathways within ECs and as a result, unique signaling pathway regulation following viral infection of the endothelium.

Activating endothelial NF-*κ*B plays a critical role in tolerance, inflammation and vascular barrier dysfunction (Sehnert et al., 2013). In ECs NF-*κ*B induces TF, endothelin-1 (ET-1), COX-2, eNOS, prostaglandins (PGI_2_/PGE_2_), PD-L1, chemokines (CXCL12, IL6,8), and inhibits TM (Friedl et al., 2002; Willoughby and Loscalzo, 2002; Sohn et al., 2005; Oganesyan et al., 2006; He et al., 2007; Pober and Sessa, 2007; Pober et al., 2009; Charoenthongtrakul et al., 2013; Lin et al., 2013). These context dependent responses control capillary tone (COX2, NO, ET-1, PGI_2_), permeability (TF/TM), immune cell recruitment and activation (chemokines, PD-L1), and platelet activation/inhibition (PGE_2_/PGI_2_/TM) that impact vascular hemostasis (Figure 1). ECs control fibrinolysis by balancing the expression of t-PA, PAI, ATIII, TM, and PAF that are dynamically regulated (Hoffman and Monroe, 2001; Willoughby and Loscalzo, 2002; Orfanos et al., 2004; Pober and Sessa, 2007; Feletou, 2011). Several of these components are associated with acute respiratory distress syndromes and are impacted by EC generated angiotensins, kinins, growth factors and integrins that control angiogenesis, platelets, capillary tone, and permeability (Pober and Sessa, 2007; Pober et al., 2009; Feletou, 2011; Taylor et al., 2013; Vaheri et al., 2013). Given the delicate balance of EC regulated hemostatic responses, an extended viral infection of endothelium alone has the potential to cause thrombocytopenia, capillary dilation/contraction and vascular permeability that are the crux of viral hemorrhagic and edematous diseases.

## VIRAL REGULATION OF PLATELETS AND PERMEABILITY

As mentioned above many factors are reported to induce permeability by acting on EC-specific or EC-related targets. HV infection of ECs alters platelet, cytokine and permeability factor responses (Mackow and Gavrilovskaya, 2009) and suggests mechanisms of EC dysregulation common to viruses that cause vascular leakage (Cosgriff et al., 1991; Zaki et al., 1995; Willoughby and Loscalzo, 2002; Olsson et al., 2003). Unique vascular EC adhesion molecules (i.e., VE-cadherin) form adherence junctions between adjacent ECs and form the primary fluid barrier of capillaries. ECs also uniquely express VEGF receptors (VEGFRs) that are activated by the binding of VEGF, a factor first described as a potent edemagenic vascular permeability factor, and controlled by a number of receptors and signaling pathways (Dvorak, 2006; Olsson et al., 2006). ECs also uniquely respond to angiopoietins 1/2 which either stabilize or enhance vascular permeability. VEGF directs localized angiogenic repair through EC dissociation and proliferation, is induced by hypoxia, contributes to high altitude induced pulmonary edema and causes chronic vascular hyperpermeability (Berger et al., 2005; Nagy et al., 2007). VEGF also induces EC expression of TF which triggers clotting and fibrinolysis that impact both platelet activation and permeability (Wood et al., 2014) and hypoxia induced PD-L1 may contribute to T cell regulation during viral infections (Noman et al., 2014).

Tissue factor was shown to be upregulated following EBOV infections of macaques suggesting that altered coagulation responses may contribute to DIC observed during EBOV infection (Geisbert et al., 2003a,b). EBOV infected ECs showed no sign of significant cytolytic effects and viral replication was not consistently seen in ECs till a day after DIC was noted (Geisbert et al., 2003b,c). Interestingly, TF activation is associated with EC permeability only when Factor VIII is also depleted, suggesting that additional EC regulated coagulation responses are likely to be altered in order to direct capillary leakage (Friedl et al., 2002; Frueh et al., 2013). Using a TF inhibitor resulted in a 33% survival rate of infected macaques and a 3 day increase in the survival time of remaining animals (Geisbert et al., 2003a). These findings suggest the potential for anti-coagulation therapy in targeting and restoring hemostasis during EBOV infections.

Pathogenic HVs bind and dysregulate α_v_β_3_ integrins on ECs that normally restrict VEGF directed permeability by binding VEGFR2 (Gavrilovskaya et al., 1999; Borges et al., 2000; Raymond et al., 2005; Coller and Shattil, 2008; Figure 1). *In vitro*, HVs coat the surface of infected ECs >3 days post-infection and render infected ECs hyperresponsive to VEGF and hypoxia directed permeability (Gavrilovskaya et al., 2008, 2013). HVs present on the EC surface bind to quiescent platelets via α_IIb_β_3_ integrins and 3 days post-infection recruit platelets to EC surfaces (Gavrilovskaya et al., 2010). These findings are consistent with HV inhibiting α_IIb_β_3_ platelet activating functions and a potential mechanism of thrombocytopenia that results in the quiescent recruitment of platelets to the surface of the HV infected endothelium. Covering the EC with a coat of quiescent platelets may also serve as a mechanism for HVs to evade immunosurveillance and suggests that HVs may induce platelet inhibitors PGI_2_/prostacyclin/TM and restrict platelet α_IIb_β_3_ integrin responses during infection of the endothelium.

## ENDOTHELIAL CELL TARGETING AND IMMUNE MEDIATED DENGUE VIRUS PERMEABILITY

Vascular leakage caused by DV infections presents a unique immune enhanced paradigm that requires preexisting anti-DV antibodies (Halstead, 2003). DVs are transmitted to man from infected mosquitoes and infect immune, dendritic and ECs (Halstead, 1988; Jessie et al., 2004; Balsitis et al., 2009; Dalrymple and Mackow, 2012b). Roles for tolerance in hosts are not rationalized in this setting and initial DV infections are typical acute RNA virus infections with little delay in onset, and flu-like symptoms predominant in DF: high fever, IFN and cytokine responses (Halstead, 2008). In contrast, DHF or DSS (systemic edema) result from an enhanced immune response following infection by a second DV serotype and occur over a 1–2 week period similar to AV, EBOV, and HV (Halstead, 1988). Vascular leakage follows the cessation of fever and virema and coincides with the late onset of thrombocytopenia which remains a central finding in DHF and DSS (Halstead, 2002; Noisakran et al., 2009) and mimics other thrombocytopenia associated diseases (Harker and Slichter, 1972; Nguyen and Carcillo, 2006).

The same cytokine responses are reported during initial and secondary DV infections, strongly suggesting that DV induced cytokines alone are insufficient to cause DHF or DSS (Halstead, 2002, 2008). DV infected ECs have been documented in several organs at autopsy (Jessie et al., 2004; Balsitis et al., 2009), and roles for ECs in DHF and DSS are without question, but poorly understood due to the invasive nature of analysis required and the relatively low mortality associated with disease. DV infection of primary human ECs is rapidly productive and elicited a constellation of chemokine responses found in patients that may foster an immune enhancing disease process and EC targeting (Halstead, 2008; Dalrymple and Mackow, 2012a,b, 2014). Initial DV infections appear to prime immune responses to crossreactive non-structural DV antigens during a second infection and thus effector T cell recruitment and immune targeting of platelets and ECs are potential causes of vascular leakage (Lei et al., 2001; Cardier et al., 2006; Chen et al., 2009). Variation in the magnitude and specificity of initial infections may impact individual DHF or DSS responses during secondary infections (Zellweger et al., 2010). The absence of animal models of DV disease and findings that DHS and DSS occur in ∼1% of human DV infections (Halstead, 2008) further complicate the development of viable disease models (Balsitis and Harris, 2010; Tan et al., 2010; Zellweger et al., 2010) and the causes of DV induced hemorrhagic and edematous disease.

## CONCLUSIONS AND DIRECTIONS

Virally induced changes in EC responses remain poorly understood but fundamental to capillary leakage, edema and hemorrhagic diseases. The likely ability of AV, EBOV, and HVs to persistently infect their hosts without disease and tolerize host immune responses provides a notable divergence between asymptomatic and pathogenic infections of the endothelium. In humans these viruses fail to establish persistence or tolerance and cause lethal hemorrhagic or edematous diseases that have in common the ability to infect the endothelium and, after long onset, cause thrombocytopenia and capillary leakage. The long prodromal period is consistent with attempted tolerance and the regulation of early immune responses that permit an extended period of EC infection. The balance of EC functions that these viruses maintain in their hosts are unsuitable for preserving human hemostasis with lethal vascular consequences. EC directed immune regulation is suggested during HV infection, while AV and EBOV infect both ECs and immune cells that may jointly defeat, tolerize or reduce early T cell clearance responses. Thrombocytopenia is caused by platelet consumption, aggregation, sequestration, or quiescence and is likely to be uniquely induced by each virus depending on the EC effector pathways and cellular receptors that are impacted. The feedback regulation and overlapping functions that maintain vascular stasis suggest that viruses alter several EC systems in order to cause hemorrhage and edema.

Currently we have little understanding of the changes that occur in virally infected ECs that might alter EC barrier functions. However, the myriad of hemostatic systems that may be altered by viral infection of the endothelium demonstrates the essential nature of defining virally induced changes in EC responses that direct hemorrhagic and edematous disease. During viral infection of ECs there is a desperate need to resolve responses that direct platelet and capillary dysfunction and reveal unique viral interactions with human EC proteins and signaling pathways. Without this information it is hard to realistically consider therapeutic targeting the intricate network of EC responses required to restore homeostasis following infection by hemorrhagic and edematous viruses.

### Conflict of Interest Statement

The authors declare that the research was conducted in the absence of any commercial or financial relationships that could be construed as a potential conflict of interest.
